# Permutation-based variance component test in generalized linear mixed model with application to multilocus genetic association study

**DOI:** 10.1186/s12874-015-0030-1

**Published:** 2015-04-22

**Authors:** Ping Zeng, Yang Zhao, Hongliang Li, Ting Wang, Feng Chen

**Affiliations:** Department of Epidemiology and Biostatistics, School of Public Health, Nanjing Medical University, Nanjing, 211166 , Jiangsu People’s Republic of China; Department of Epidemiology and Biostatistics, Center of Medical Statistics and Data Analysis, School of Public Health, Xuzhou Medical College, Xuzhou, 221004 Jiangsu People’s Republic of China; Center for Disease Control and Prevention of Pudong New Area, Pudong New Area, Shanghai, 200136 People’s Republic of China

**Keywords:** Likelihood ratio test, Permutation procedure, Variance component, Multilocus association analysis, Working response, Penalized quasi-likelihood algorithm, Generalized linear mixed model, Logistic mixed effects model

## Abstract

**Background:**

In many medical studies the likelihood ratio test (LRT) has been widely applied to examine whether the random effects variance component is zero within the mixed effects models framework; whereas little work about likelihood-ratio based variance component test has been done in the generalized linear mixed models (GLMM), where the response is discrete and the log-likelihood cannot be computed exactly. Before applying the LRT for variance component in GLMM, several difficulties need to be overcome, including the computation of the log-likelihood, the parameter estimation and the derivation of the null distribution for the LRT statistic.

**Methods:**

To overcome these problems, in this paper we make use of the penalized quasi-likelihood algorithm and calculate the LRT statistic based on the resulting working response and the quasi-likelihood. The permutation procedure is used to obtain the null distribution of the LRT statistic. We evaluate the permutation-based LRT via simulations and compare it with the score-based variance component test and the tests based on the mixture of chi-square distributions. Finally we apply the permutation-based LRT to multilocus association analysis in the case–control study, where the problem can be investigated under the framework of logistic mixed effects model.

**Results:**

The simulations show that the permutation-based LRT can effectively control the type I error rate, while the score test is sometimes slightly conservative and the tests based on mixtures cannot maintain the type I error rate. Our studies also show that the permutation-based LRT has higher power than these existing tests and still maintains a reasonably high power even when the random effects do not follow a normal distribution. The application to GAW17 data also demonstrates that the proposed LRT has a higher probability to identify the association signals than the score test and the tests based on mixtures.

**Conclusions:**

In the present paper the permutation-based LRT was developed for variance component in GLMM. The LRT outperforms existing tests and has a reasonably higher power under various scenarios; additionally, it is conceptually simple and easy to implement.

## Background

### LRT for variance component in LMM

In many medical researches the main interest often lies on the problem of testing whether a random effects variance component is equal to zero within the mixed effects models framework. The variance component test has long been a statistical challenge and received much attention in the literature; see for example, Self and Liang [1], Stram and Lee [2], Liang and Self [3], more recently Lindquist, et al. [4], Drikvandi, et al. [5], Nobre, et al. [6], and many others. Beyond the direct interest, some other scientific problems under investigation can be also transformed into this similar issue. For example, to test a parametric null model against a nonparametric alternative in the penalized spline regression, Claeskens [7] first constructed a mixed effects model so that the hypothesis testing was reduced to the problem that whether the variance component of random effects was zero, and then performed a restricted likelihood ratio lack-of-fit test. Analogous transforms and hypothesis testing problems were also studied by Crainiceanu and Ruppert [8-10], Crainiceanu, et al. [11] and Greven, et al. [12].

The variance component test is nonstandard in the sense that under the null the variance component is on the boundary of the parameter space. In this situation it is not easy to obtain the null distribution of the likelihood ratio statistic. Under some regularity conditions Self and Liang [1], Stram and Lee [2] and Liang and Self [3] proved that the likelihood ratio statistic followed a 0.50:0.50 mixture of ${\chi}_0^2$ and ${\chi}_1^2$, where ${\chi}_0^2$ is a point mass at zero and ${\chi}_1^2$ is a chi-square distribution with one degree of freedom. Whereas Crainiceanu and Ruppert [8] argued that the assumed conditions in these papers were often not guaranteed in practice, they showed the mixture proportion parameter is actually dependent on specific contexts and the equal-weight mixture can lead to conservative type I error control. To obtain the null distribution of the likelihood ratio test (LRT) statistic, Crainiceanu and Ruppert [8] developed a simulation-based algorithm using the spectral representation of the LRT statistic.

Instead of using the equal-weight mixture as done in Self and Liang [1] and Liang and Self [3], Pinheiro and Bates [13] found that a nonequal-weight one may be better and suggested the 0.65:0.35 mixture for some specific longitudinal datasets. Fitzmaurice, et al. [14] evaluated the 0.50:0.50 and 0.65:0.35 mixtures via simulations and concluded that the appropriate mixture is not straightforward to derive.

The work aforementioned shows clearly that it is difficult to obtain an analytical expression for the null distribution of the likelihood ratio statistic. On the other hand, when encountering complex hypothesis testing scenarios in practical data analyses, resorting to resampling-based methods is a very natural and effective strategy. Faraway [15] and Samuh, et al. [16] applied the parametric bootstrap approach for testing the variance component. Lee and Braun [17] and Samuh, et al. [16] used the permutation procedure to resolve this problem. Their results demonstrated that the bootstrap and permutation tests can control the type I error rate correctly and are more powerful compared to the tests that are based on the usual asymptotic mixture.

### LRT for variance component in GLMM

At present most of the work concerning the likelihood-ratio based variance component test has been mainly investigated under the context of linear mixed models (LMM), in which the response variable is continuous and the closed-form log-likelihood function is easily obtained and calculated [18,19]. However, little literature has been published about LRT of variance component in the generalized linear mixed models (GLMM) framework [20,21], where the response variable is discrete, such as the binary or count variable, and the calculation of the log-likelihood function is not straightforward.

The difficulty of performing the likelihood ratio variance component test in GLMM arises in several aspects: (*i*) unlike in LMM for the continuous data, the closed-form log-likelihood function of GLMM can be no longer yielded except in some extremely limited situations, thus the computation of the likelihood ratio statistic is not easy; (*ii*) the exact parameter estimation in GLMM is often impossible because of the need of high-dimensional numerical integration; (*iii*) the null distribution of the likelihood ratio statistic in GLMM is complicated, and similar to the case in LMM it cannot be obtained analytically.

Lee and Braun [17] mentioned in their Discussion Section that the permutation test can be applied to the problem of testing for variance components in GLMM, but did not formally give any further results to date. Fitzmaurice, et al. [14] investigated the permutation variance component test in GLMM to detect the cluster effect in a multilevel binary dataset and showed that the permutation test can effectively control the type I error rate and has a higher power compared with the tests based on the asymptotic mixture of chi-square distributions.

Lin [22] developed a global score test to examine variance components in GLMM via the manner of Taylor expansion of the integrated quasi log-likelihood, derived the analytical null distribution of the score statistic, and displayed that this score test was a locally asymptotically most powerful test under some assumptions; see also Zhang and Lin [23] for similar presentations where the score test was constructed for the semi-parametric generalized additive mixed models [24]. Sinha [25] constructed a score-type statistic for the variance component test in GLMM via parametric bootstrap procedure and showed that the bootstrap score test was valid.

### A motivating application for the variance component test in GLMM

In this paper we attempt to perform the likelihood ratio variance component test in GLMM via the permutation procedure. To present the permutation-based LRT, we now give an example motivating from genetic statistics concerning multilocus association analyses in case–control studies. One of the main objectives in genetic association studies is to identify the causal markers (e.g., single nucleotide polymorphisms, SNPs) that are significantly related to diseases. Two commonly used methods in genome-wide association studies are respectively single marker analysis and multiple markers analysis [26]. Compared with the single marker analysis, the multiple markers analysis is generally more powerful since some useful information among markers is taken into account.

More specifically, suppose that in multilocus association analyses *K* SNPs within a functional gene are grouped into an SNP set. The objective is to test whether these *K* SNPs are jointly associated with the disease of interest, say y. Here we assume y is a binary variable. If the number of SNPs (i.e., *K*) is small, we can perform traditional fixed effects model and test to achieve this aim; however, if the number of SNPs is large, the traditional test such as the Wald test or the score test is typically underpowered for the null due to the large number of degrees of freedom, and the fixed effects model may be subject to some numerical issues, e.g., the problem of multicollinearity since the SNPs within a gene may be highly correlated due to the linkage disequilibrium.

As an effective alternative, if the effects of SNPs are assumed to be random with a common variance component [27], then the fixed effects model becomes the mixed effects model and the test is converted into the variance component test under the framework of GLMM (i.e., the logistic mixed effects model). And now the numerical problems encountered in the fixed effects model mentioned above can be avoided because of the use of mixed effects model [27-31].

It should be emphasized that our main objective is focused on the hypothesis testing of variance component in GLMM instead of the parameter estimation. In this paper we attempt to explore the application of LRT in logistic mixed effects model based on the well-developed theories for GLMM.

The organization of this paper is given as follows. The likelihood ratio statistic is constructed by taking full of the working response generated in the estimation process of GLMM. To circumvent the derivation of the analytical expression for the null distribution of the likelihood ratio statistic, we use the permutation procedure. We implement simulation studies to evaluate the performance of the proposed permutation-based LRT in the context of genetic association studies. Finally we apply the proposed LRT to multilocus association analysis for the binary genetic data.

## Methods

### Logistic mixed effects model and the variance component test

Let **X** = [*X*_0_, *X*_1_, …, *X*_*p*_] and **Z** = [*Z*_1_, *Z*_2_, …, *Z*_*K*_] be *n* × (*p +* 1) and *n* × *K* matrices for covariates, respectively, where *X*_0_ = 1 corresponds to the intercept term, *n* is the total sample size; and let **y** = [y_1_, y_2_, …, y_*n*_]’ be a *n*-dimensional vector for a binary response variable coded as 0 and 1, such as y = 0 for the control and y = 1 for the case in case–control studies. Assume the relationship between **y**, **X** and **Z** is characterized via the following logistic model

1 $$ \begin{array}{l}\kern6.5em \boldsymbol{\mu} \kern1em =\kern0.5em E\left(\mathbf{y}\right),\\ {} \log \left(\frac{\boldsymbol{\mu}}{1\kern0.5em -\kern0.5em \boldsymbol{\mu}}\right)\kern0.5em =\kern0.5em {\displaystyle \sum_{j\kern0.5em =\kern0.5em 0}^p{X}_j{\alpha}_j}\kern0.5em +\kern0.5em {\displaystyle \sum_{k\kern0.5em =\kern0.5em 1}^K{Z}_k{\beta}_k}\kern0.5em =\kern0.5em \mathbf{X}\boldsymbol{\alpha } \kern0.5em +\kern0.5em \mathbf{Z}\boldsymbol{\beta }, \\ {}\kern6em {\beta}_k\kern0.5em \sim \kern0.5em N\left(0,\kern0.5em {\tau}^2\right),\end{array} $$

where ***α*** = [*α*_0_, *α*_1_, …, *α*_*p*_] are considered as fixed effects, while ***β*** = [*β*_1_, *β*_2_, …, *β*_*K*_] are considered as random effects following a normal distribution with mean zero and variance component *τ*^2^. Under these settings model (1) becomes the logistic mixed effects model which has been widely studied in the literature [20,21,32,33]. Our aim is to test *β*s simultaneously to determine the overall significance of variables **Z**. Based on these specifications, the test of ***β*** = 0 is immediately equivalent to the test of variance component *H*_0_: *τ*^2^ = 0 in model (1).

Note that model (1) is different from the one considered by Fitzmaurice, et al. [14] and Sinha [25], in which only one random effect was included so that the approximated calculation of the log-likelihood function via numerical integration was feasible; whereas in model (1) there are *K* random effects with the same variance component and the calculation of the log-likelihood function via numerical integration is generally not possible [21].

### Definition of the likelihood ratio statistic

A lot of algorithms have been developed for estimating GLMM, including approximate approaches and Monte Carlo methods [20,21,34]. Here we use the penalized quasi-likelihood (PQL) algorithm [20,34] since it has the conceptual and computational advantage compared to others and can be implemented via existing software, such as the glmmPQL function in the R package MASS [35].

We build LRT for the null of *H*_0_: *τ*^2^ = 0 in model (1) by using the working response variable, denoted as ***Y****’* to distinguish the original response variable **y**, generated after the convergence of PQL algorithm

2 $$ {\boldsymbol{Y}}^{\boldsymbol{\prime}}\kern0.5em =\kern0.5em \mathbf{X}\boldsymbol{\alpha } \kern0.5em +\kern0.5em \mathbf{Z}\boldsymbol{\beta } \kern0.5em +\kern0.5em \boldsymbol{e},\kern0.5em \beta \kern0.5em \sim \kern0.5em N\left(0,\kern0.5em {\tau}^2\right),\kern0.5em \boldsymbol{e}\kern0.5em \sim N\left(0,\kern0.5em \mathbf{R}\right), $$

where **R** is a *n* × *n* diagonal matrix with elements being 1/[*μ*(1-*μ*)]. Following Breslow and Clayton [20], the corresponding quasi log-likelihood up an unrelated constant for the working response variable ***Y****’* is

3 $$ L\left({\tau}^2\right)\kern0.5em \approx \kern0.5em -\frac{1}{2}\left[ \log \left|\mathbf{V}\right|\kern0.5em +\kern0.5em {\left({\boldsymbol{Y}}^{\boldsymbol{\prime}}\kern0.5em -\mathbf{X}\widehat{\boldsymbol{\alpha}}\right)}^{\prime }{\mathbf{V}}^{-1}\left({\boldsymbol{Y}}^{\boldsymbol{\prime}}\kern0.5em -\mathbf{X}\widehat{\boldsymbol{\alpha}}\right)\right], $$

with **V** = *τ*^2^**ZZ**′ + **R** and $\widehat{\boldsymbol{\alpha}}\kern0.5em =\kern0.5em {\left({\mathbf{X}}^{\mathbf{\prime}}{\mathbf{V}}^{-1}\mathbf{X}\right)}^{-1}{\mathbf{X}}^{\mathbf{\prime}}{\mathbf{V}}^{-1}{\boldsymbol{Y}}^{\boldsymbol{\prime}}.$ By carefully inspecting we can easily find that Formula (3) is actually the log-likelihood function of LMM with residual term ***ε*** and random effects ***β*** if ***Y****’* is treated as a continuous response variable. Based on the log-likelihood given in (3), the likelihood ratio statistic for *H*_0_: *τ*^2^ = 0 can be defined as

4 $$ T\kern0.5em =\kern0.5em 2\left[{ \sup}_{\tau^2\kern0.5em \ge \kern0.5em 0}L\left({\tau}^2\right)\kern0.5em -\kern0.5em L\left({\tau}^2\kern0.5em =\kern0.5em 0\right)\right]. $$

Here we have some remarks about the LRT statistic given in (4): (*i*) we calculate *T* via the working response variable ***Y****’* instead of the original response variable **y**; (*ii*) for fair comparisons, the log-likelihood of the null model, i.e., the value of *L*(*τ*^2^ = 0), is also calculated according to the quasi log-likelihood, although the null model with *τ*^2^ = 0 reduces to the general logistic regression whose log-likelihood can be computed directly; in fact we find that negative values of the likelihood ratio statistic are obtained if the log-likelihood of the reduced logistic regression is adopted; more importantly, we indeed do not see any problems with the use of Formula (4) to compute the likelihood ratio statistic in our simulations; (*iii*) here we only consider LRT, although the restricted likelihood ratio test [8-10] can be also applied; the difference between the two likelihood ratio tests is generally ignorable as the sample size increases.

### Resampling algorithm for the null distribution of *T*

To obtain the null distribution of the likelihood ratio statistic *T*, we now use the permutation procedure [36-38]. The more general resampling algorithm is described as follows.

- ***Resampling algorithm for the null distribution of T***
- *step* 1: Calculate the observed value of *T* according to (4) using the original data **D** = [**y**, **X**, **Z**], say *T*^obs^;
- *step* 2: Generate a new dataset D^*^ by resampling;
- *step* 3: Calculate the new value of *T* according to (4) using D^*^, say T^*^;
- *step* 4: Repeat the steps 2–3 above *B* times, get T^*b^, *b* = 1, 2, …, *B*;
- *step* 5: The resampling-based null distribution of *T* can be approximated by T^*b^, and the Monte Carlo p value of *T* is estimated by the proportion of T^*b^ equal to or greater than *T*^obs^.

In step 2 of the resampling algorithm, if we randomly permute **y** and **X** as a whole while keeping **Z** fixed, then we are performing the permutation test [14,38]. Here we implicitly assume that no correlation between **X** and **Z** exists. If we only generate the new response variable **y** (say y*) from the fitted null model (i.e., the fixed effects logistic model with only covariates **X**) while letting both **X** and **Z** unchanged, then we are performing the parametric bootstrap test [15,16,25,36,37]. The permutation and bootstrap algorithms are almost the same except the production of the new resampling dataset in the second step. In this paper we only discuss the permutation test since it requires fewer assumptions compared with the parametric bootstrap test.

Usually, the number of replicates *B* in the permutation test is set to 1000 ~ 2000 for a relatively large significance level, say *α* = 0.05. Whereas some authors [14,16] empirically found that much smaller values of *B* (e.g., 200 or 500) could also behave satisfactorily.

### Simulation study and real application

In this section we implemented simulation studies to evaluate the proposed permutation-based LRT method and then applied it to the real genetic data. The simulation data was generated via the following logistic model

5 $$ \log \left[\frac{E\left(\mathbf{y}\right)}{1\kern0.5em -\kern0.5em E\left(\mathbf{y}\right)}\right]\kern0.5em =\kern0.5em -\left(1\kern0.5em +\kern0.5em 0.5{X}_1\kern0.5em +\kern0.5em 0.5{X}_2\right)\kern0.5em +\kern0.5em {\displaystyle \sum_{k\kern0.5em =\kern0.5em 1}^K{Z}_k}{\beta}_k, $$

where *X*_1_ is a binary variable with a probability of 0.5 and *X*_2_ is a standard normal variable, and they are mutually independent. To correspond to the motivating example of multilocus association studies described before and to mimic the true genetic data, in this paper we use the additive genetic model so that *Z* = 0, 1 and 2 represents the count of the minor allele [31].

For comparisons, besides the proposed permutation-based LRT, the methods of LRT based on the 0.50:0.50 and 0.65:0.35 mixtures and the score-based variance component test are also implemented. It should be mentioned that the 0.65:0.35 mixture suggested by Pinheiro and Bates [13] is only reasonable for some specific datasets and may not apply to the present simulation settings; but it has been shown that it provided a useful reference in some cases [8,14], thus it is still considered here. The score variance component tests in mixed effects models have been previously investigated by many authors in a wide range of application areas [22,23,28-31,39]; these score tests share a very similar rationale to each other. In this paper we use the score test originally developed by Lin [22], which was later applied and called sequence kernel association test (SKAT) by Wu, et al. [31] under the context of rare variants association in sequencing data and is implemented in the R package SKAT (version 0.91) [40]. All the data analyses are performed within the R (version 2.15.3) statistical environment [41].

The sample size was set to 400, 600, 800 and 1000. The value of *B* in the permutation-based LRT was 1000. The number of simulations was 2000 for controlling the type I error rate and was 1000 for evaluating the statistical power. The estimated type I error rate and power were calculated as the proportion of the p-values less than the given significance level α = 0.05.

### Simulation 1

We first evaluated the type I error and power of the proposed LRT in the context of multilocus association analyses with different number of SNPs and various random effects variance component. To obtain Z = [*Z*_1_, *Z*_2_, . . ., *Z*_*K*_], we first generated G = [*G*_1_, *G*_2_, . . ., *G*_*K*_] via the standard normal distribution with pairwise correlation between *G*_*k*_ and *G*_*k*’_ to be 0.5^|*k* - *k*’|^, and then set *Z*_*k*_ = 0, 1 and 2 according to whether *G*_*k*_ *< −c*, −*c* ≤ *G*_*k*_ *≤ c* or *G*_*k*_ > *c*, where *c* is the third quartile of the standard normal distribution. We set *K* = 20, 30 and 40, and *β* = 0 in model (5) for the type I error simulation and sampled *β* from a normal distribution with mean zero and variance *τ*^2^ = 0.15 and 0.20 for the power evaluation. The results are given in Tables 1 and 2.

**Table 1:** **Estimated type I error rates for simulation 1 with varying number of random effects**

***n***	**Method**	***K***		
		**20**	**30**	**40**
400	Permutation	0.046	0.055	0.054
	Score	0.043	0.045	0.048
	Mixture (0.50)	0.016	0.020	0.021
	Mixture (0.65)	0.020	0.026	0.027
600	Permutation	0.052	0.047	0.044
	Score	0.045	0.042	0.037
	Mixture (0.50)	0.016	0.016	0.013
	Mixture (0.65)	0.020	0.021	0.017
800	Permutation	0.052	0.054	0.051
	Score	0.048	0.052	0.047
	Mixture (0.50)	0.014	0.015	0.012
	Mixture (0.65)	0.020	0.019	0.020
1000	Permutation	0.051	0.041	0.053
	Score	0.050	0.036	0.051
	Mixture (0.50)	0.017	0.014	0.011
	Mixture (0.65)	0.020	0.017	0.017

*Note:* Permutation is the proposed permutation-based LRT, Score is the score-based sequence kernel association test given in Wu, et al. [31] that was originally developed in Lin (1997), Mixture(0.50) and Mixture (0.65) respectively correspond to the asymptotic 0.50:0.50 and 0.65:035 mixtures of chi-square distributions. Here *K* is the number of random effects, i.e., the number of SNPs included in a gene.

**Table 2:** **Estimated powers for Simulation 1 with the random effects variance component equal to 0.15 and 0.20 and varying number of random effects**

***n***	**Method**	***K*** **with** ***τ*** ^**2**^ ***=*** **0.15**			***K*** **with** ***τ*** ^**2**^ ***=*** **0.20**		
		**20**	**30**	**40**	**20**	**30**	**40**
400	Permutation	0.296	0.380	0.433	0.506	0.577	0.704
	Score	0.276	0.363	0.402	0.501	0.557	0.689
	Mixture (0.50)	0.158	0.235	0.282	0.349	0.441	0.567
	Mixture (0.65)	0.187	0.269	0.318	0.393	0.476	0.615
600	Permutation	0.436	0.566	0.630	0.712	0.837	0.878
	Score	0.430	0.548	0.604	0.704	0.816	0.868
	Mixture (0.50)	0.255	0.382	0.463	0.538	0.686	0.794
	Mixture (0.65)	0.292	0.437	0.501	0.589	0.717	0.826
800	Permutation	0.559	0.704	0.777	0.840	0.922	0.951
	Score	0.557	0.702	0.771	0.830	0.917	0.948
	Mixture (0.50)	0.380	0.526	0.642	0.727	0.851	0.905
	Mixture (0.65)	0.427	0.573	0.682	0.765	0.873	0.922
1000	Permutation	0.688	0.792	0.865	0.913	0.950	0.987
	Score	0.679	0.787	0.864	0.909	0.949	0.983
	Mixture (0.50)	0.517	0.637	0.774	0.828	0.914	0.966
	Mixture (0.65)	0.562	0.677	0.801	0.852	0.926	0.970

*Note:* Permutation is the proposed permutation-based LRT, Score is the score-based sequence kernel association test given in Wu, et al. [31] that was originally developed in Lin (1997), Mixture (0.50) and Mixture (0.65) respectively correspond to the asymptotic 0.50:0.50 and 0.65:035 mixtures of chi-square distributions. Here *K* is the number of random effects, i.e., the number of SNPs included in a gene, and *τ* ^2^ is the random effects variance component.

### Simulation 2

This simulation is mainly designed to see the influence of non-normally distributed random effects in multilocus association studies. Here the variable Z in model (5) was generated via the software COSI [42] based on a coalescent model for European population. Specifically, we first generated a long region with COSI, from which a continuous subregion was selected so that it contained a reasonable number of SNPs; next this subregion was treated as a gene, whose genotypes were specified to *Z*, i.e., the SNPs included in this subregion are modeled by variables *Z*. The average number of SNPs included in the simulation is given in Table 3. Usually, not all of the SNPs (i.e., Z) within a gene are causal [31], thus some of them were assumed to have zero effects; i.e., only *m* out of *K* SNPs had an impact on the response variable y in model (5). Here we set *m* = 0 *K*, 0.1 *K*, 0.3 *K* and 0.5 *K*. The effects ***β*** were generated from the uniform distribution *U*[0.60, 1.10] at random. The results are given in Table 3.

**Table 3:** **Estimated powers for Simulation 2 with different proportion of the causal markers**

***n***	**Method**	***K***	**Power with various** ***m***			
			**0** ***K***	**0.1** ***K***	**0.3** ***K***	**0.5** ***K***
400	Permutation	46	0.052	0.112	0.252	0.402
	Score		0.046	0.103	0.241	0.396
	Mixture (0.50)		0.042	0.096	0.226	0.371
	Mixture (0.65)		0.066	0.130	0.262	0.414
600	Permutation	54	0.053	0.138	0.361	0.583
	Score		0.046	0.125	0.345	0.576
	Mixture (0.50)		0.046	0.121	0.329	0.550
	Mixture (0.65)		0.063	0.153	0.388	0.604
800	Permutation	60	0.049	0.181	0.446	0.707
	Score		0.040	0.173	0.425	0.685
	Mixture (0.50)		0.039	0.149	0.406	0.669
	Mixture (0.65)		0.057	0.192	0.471	0.721
1000	Permutation	65	0.051	0.181	0.528	0.754
	Score		0.046	0.178	0.513	0.746
	Mixture (0.50)		0.040	0.159	0.492	0.727
	Mixture (0.65)		0.057	0.193	0.552	0.773

*Note:* Permutation is the proposed permutation-based LRT, Score is the score-based sequence kernel association test given in Wu, et al. [31] that was originally developed in Lin (1997), Mixture (0.50) and Mixture (0.65) respectively correspond to the asymptotic 0.50:0.50 and 0.65:035 mixtures of chi-square distributions. Here *K* is the number of random effects, i.e., the number of SNPs included in a gene, and *m* is the number of causal SNPs; when *m* = 0 (i.e., corresponding to 0 *K* in the fourth column), the estimated power is actually the type I error rate.

### Data analysis

We applied the permutation-based LRT to the well-known Genetic Analysis Workshop 17 (GAW17) mini-exome sequencing data [43], which was constructed based on the 1000 Genomes Project [44]. The GAW17 data contain a binary response variable called *affection status* (say y here, and y = 1 representing affection and y = 0 representing non-affection) and five covariates including *age* (*X*_1_, a continuous variable), *smoking status* (*X*_2_, an indicator variable representing smoking or non-smoking), and three continuous variables *Q*_1_, *Q*_2_ and *Q*_4_. The number of individuals is 697. The details of this data were described in Almasy, et al. [43]. We implemented multilocus association analysis for four genes *ELAVL4*, *PRKCB1*, *PTK2B* and *SOS2* across all the 200 replicates of the GAW17 data.

The logistic mixed effects model for the GAW17 data is written as

6 $$ \begin{array}{l} \log \left[\frac{E\left(\mathrm{y}\right)}{1\kern0.5em \hbox{-} \kern0.5em E\left(\mathrm{y}\right)}\right]\kern0.5em =\kern0.5em {\alpha}_0\kern0.5em +\kern0.5em {\alpha}_1\kern0.5em \times \kern0.5em {X}_1\kern0.5em +\kern0.5em {\alpha}_2\kern0.5em \times \kern0.5em {X}_2+\kern0.5em {\alpha}_3\kern0.5em \times \kern0.5em {Q}_1\kern0.5em +\kern0.5em {\alpha}_4\kern0.5em \times \kern0.5em {Q}_2\kern0.5em +\kern0.5em {\alpha}_5\kern0.5em \times \kern0.5em {Q}_4\kern0.5em +\kern0.5em {\displaystyle \sum_{k\kern0.5em =\kern0.5em 1}^K{Z}_k{\beta}_k},\\ {}\kern8.5em {\beta}_k\kern0.5em \sim \kern0.5em N\left(0,\kern0.5em {\tau}^2\right),\end{array} $$

here *Z* is the genotype of the selected gene, and *α*_0_*- α*_5_ are set to be fixed effects while ***β*** are set to be random effects. Our interest is to test *H*_0_: *τ*^2^ = 0 in model (6) to determine whether a given gene is associated with *affection status*. Since in this data we know which SNPs are causal, the proportion of p-values less than the given significance level provides a measurement of the empirical power for these methods. The results are given in Table 4.

**Table 4:** **Results of the four genes in GAW17 data**

**Gene**	**Method**	***K***	***m***	**The significance level** ***α***		
				**0.05**	**0.01**	**0.001**
*ELAVL4*	Permutation	10	2	0.725	0.630	0.395
	Score			0.450	0.240	0.040
	Mixture (0.50)			0.640	0.485	0.335
	Mixture (0.65)			0.690	0.540	0.360
*PTK2B*	Permutation	18	3	0.600	0.470	0.290
	Score			0.085	0.030	0.000
	Mixture (0.50)			0.515	0.355	0.245
	Mixture (0.65)			0.540	0.405	0.255
*PRKCB1*	Permutation	20	1	0.550	0.355	0.255
	Score			0.095	0.030	0.005
	Mixture (0.50)			0.430	0.305	0.220
	Mixture (0.65)			0.465	0.315	0.225
*SOS2*	Permutation	9	2	0.545	0.400	0.235
	Score			0.160	0.030	0.005
	Mixture (0.50)			0.420	0.285	0.165
	Mixture (0.65)			0.450	0.325	0.185

*Note:* Permutation is the proposed permutation-based LRT, Score is the score-based sequence kernel association test given in Wu, et al. [31] that was originally developed in Lin (1997), Mixture (0.50) and Mixture (0.65) respectively correspond to the asymptotic 0.50:0.50 and 0.65:035 mixtures of chi-square distributions. In the last three columns of the table are the proportion of p-values less than *α* among the 200 replicates. Here *K* is the number of SNPs included in the gene, i.e., the number of the random effects contained in the logistic mixed effects model (5) and *m* is the number of causal SNPs.

To reduce computation time of the permutation-based LRT, the simulations and data analyses were conducted with the high performance computing at the School of Public Health of Nanjing Medical University. More specifically, we use the function %dopar% in the R package doMC (version 1.3.3) [45] with the function registerDoMC having its argument cores = 30 for parallel execution when implementing the permutation procedure. Our experience showed that this parallel execution could substantially reduce the computation compared to the use of the simple R for-loop.

## Results

From Table 1 it is observed that the type I error rate of the permutation-based LRT is very close to the nominal significance level 0.05 at various situations. On the other hand, the score test is sometimes slightly conservative, especially when the sample size is relatively small (e.g., 400 and 600); the two tests respectively based on the 0.50:0.50 and 0.65:0.35 mixtures cannot control the type I error rate correctly regardless of the sample size and the number of random effects, and are much more conservative than the score test.

Table 2 shows that the permutation-based LRT is the most powerful method among these tests and the score test has higher power compared to the tests based on mixtures. Typically, as expected the powers of these tests improve as the sample size increases and the number of random effects becomes larger. In Simulation 1 as the sample size improves, the powers of the LRT and the score test approach each other. Here an interesting finding in Table 2 is that the magnitude of the random effects variance component *τ*^2^ has a greater influence on the statistical power for these tests than the number of random effects (i.e., *K*). For example, in Table 2 when *K* becomes from 20 to 40 and *τ*^2^ *=* 0.15, the increasing power for LRT is 0.137; when *K* = 20 and *τ*^2^ increases from 0.15 to 0.20, the corresponding change of power of LRT is 0.210.

In Table 3 for Simulation 2 where the random effects are not normally distributed, as before the permutation test holds the type I error rate effectively while the score test is slightly conservative and the tests based mixtures give incorrect control. For instance, the type I error rate estimated from the 0.50:0.50 mixture is conservative and the type I error rate from the 0.65:0.35 mixture is liberal, and the incorrect control of the two mixtures does not improve with the increasing sample size. The power of the permutation-based LRT is higher than that obtained from the 0.50:0.50 mixture and lower than that obtained from the 0.65:0.35 mixture. But here only the results of the LRT are valid and believable because the two mixtures cannot correctly control the type I error rate as demonstrated.

In general, as expected the powers of these tests improve as the proportion of causal markers increases. In Table 3 we find that the permutation-based LRT always outperforms the score test even when the sample size is large (e.g., 1000 and 800). In conclusion in this simulation we see that the proposed LRT can maintain a reasonably higher power than the score test even if the distribution of random effects is not normal.

From Table 4 we can see that the permutation-based LRT has higher chance to detect the causal genes compared the other three methods. Here it is somewhat surprising that the score test is extremely underpowered and has the lowest probability to identify the associated genes. For example, when the significance level is 0.001 the chance that these four genes are deemed to be statistically related to the disease status by the score test is less than 5%; relatively, the LRT has a much larger probability than the score test.

As a reviewer pointed out that, in fact, the GAW17 data are not purely real data but rather 200 replicates, which were simulated independently based on the true genotype-phenotype association model with the same genotypes but different phenotypes. Therefore, it may be limited for evaluating the performance of these tests. Nevertheless, according to the results presented in Table 4, it seems to be reasonable to conclude that the proposed permutation-based LRT has an empirical advantage to identify association signals compared with other competing tests.

## Discussion

In this paper we have applied the LRT to GLMM by taking full use of the PQL algorithm and the resulting working response variable. The main difficulties of using LRT in GLMM are the computation of the log-likelihood function for the alternative model (e.g., the logistic mixed effects model) and the derivation of the null distribution of the likelihood ratio statistic. To avoid the direct computation we use the quasi log-likelihood of the working response, which can be deemed to be linear. By doing this the LRT in GLMM becomes computationally feasible. To obtain the null distribution of LRT we use the permutation procedure which has been extensively employed in practice and has proved to be valid [14,17,38].

In the present paper we only consider the permutation test for the likelihood inference, but extending to other resampling-based methods is straightforward; for example, the parametric bootstrap method. Still the permutation test has an advantage that it requires few model assumptions compared to other resampling-based methods [14,17,38]. Via simulations it has been shown that the permutation-based LRT can effectively control the type I error rate under different situations. Similar to the results demonstrated by others [8,14,17], the usually-used asymptotic mixture distributions such as the 0.50:0.50 and 0.65:0.35 mixtures cannot maintain the type I error rate correctly at the nominal significance level. The simulations show that the score test can control the type I error rate but is sometimes conservative, especially for small sample sizes; the similar result has also been observed by Wu, et al. [31] and Li, et al. [46] under the context of multilocus association studies.

Our studies have shown that the permutation-based LRT has higher power than the score test and the tests based on mixtures under a wide range of scenarios and it still maintains a reasonably high power even when the random effects do not follow a normal distribution. The LRT has the advantage that it is conceptually simple and easy to implement, but the permutation-based LRT is computationally intensive. However, as Lee and Braun [17] pointed out that the computation burden can be reduced significantly via parallel computing; additionally, some approximation methods suggested in linear mixed effects models may be applied [8,12]. In contrast, the tests based on mixtures are relatively rapid since they do not require resampling, and the score test is the most computationally efficient since it only needs to fit the null model and its null distribution can be obtained analytically [22-24]. Another advantage of the score test is that its statistic is calculated much more straightforward [22-24].

The computation time for the permutation-based LRT depends on the sample size, the number of random effects and the number of replicates *B* in the permutation algorithm. Although having been performed the parallel computation, we still find that the permutation-based LRT is much slower than other methods, especially than the score test. For example, in Simulation 1 when the sample size is 400 and *B* = 1000 in the permutation-based LRT, running one simulation requires respectively 975 s, 702 s and 1217 s if *K* = 20, 30 and 40; whereas the score test only needs about 16 s, 23 s and 30 s if running ten simulations. All the times are obtained via the use of the function %dopar% as mentioned before.

Besides the increasing computational burden of the permutation-based LRT, another disadvantage is that the LRT may be numerically unstable to fit the alternative model when the sample size is limited and the number of the random effects is large. The third shortcoming of the LRT is that its validity and efficiency rely largely on the PQL algorithm, which had been already proved to be seriously biased downward [20,22,47,48]. Although no negative effect on the type I error controlling is observed in our simulations, certainly it can result in power reduction due to the biased estimation. Therefore, designing more efficient and faster algorithms for the proposed LRT is necessary in the future, and developing likelihood-ratio based variance component tests in GLMM with the bias-corrected PQL estimation is another interesting problem.

Finally, we note in Table 4 for GAW17 data analysis that the overall power for these methods compared in this paper is very low. For example, if we set the significance level at 0.001, none of these methods has an empirical power greater than 0.50. The reasons may be that: (*i*) the effects of the markers are very weak; (*ii*) the non-causal SNPs included in the gene lead to the reduction of power; that is, not all the random effects have nonzero coefficients; (*iii*) the sample size is relatively small for identifying the association signal; (*iv*) in the GAW17 data most of the SNPs are rare variants, i.e., their minor allele frequencies are very low and typically less than 0.01 [43], which are known to have an extremely limited power to be detected [31,49]. These results also suggest that developing more powerful methods for the low-frequency variants in case–control sequence data is an urgent demand [31,50-52].

## Conclusions

In the present paper the permutation-based LRT was developed for variance component in GLMM. Via simulations and real application it has been demonstrated that the developed LRT method outperforms the existing tests and has a reasonably high power under various scenarios; additionally it is conceptually simple and easy to implement.

## Abbreviations

LRT: Likelihood ratio test
GLMM: Generalized linear mixed model
LMM: Linear mixed model
GWAS: Genome-wide association study
PQL: Penalized quasi-likelihood
GAW17: Genetic analysis workshop 17
SNP: Single nucleotide polymorphism
